# Psychosocial Factors in Adolescence and Risk of Development of Eating Disorders

**DOI:** 10.3390/nu14071481

**Published:** 2022-04-01

**Authors:** Fernando Mora, Miguel A. Alvarez-Mon, Sonia Fernandez-Rojo, Miguel A. Ortega, Miriam P. Felix-Alcantara, Isabel Morales-Gil, Alberto Rodriguez-Quiroga, Melchor Alvarez-Mon, Javier Quintero

**Affiliations:** 1 Department of Psychiatry and Mental Health, Hospital Universitario Infanta Leonor, 28031 Madrid, Spain; fernando.mora@salud.madrid.org (F.M.); sfernandezr@salud.madrid.org (S.F.-R.); miriampatricia.felix@salud.madrid.org (M.P.F.-A.); alberto_rodriguezquiroga@yahoo.com (A.R.-Q.); fjquinterog@salud.madrid.org (J.Q.); 2 Department of Legal Medicine and Psychiatry, Complutense University, 28040 Madrid, Spain; miguel.angel.ortega92@gmail.com; 3 Department of Medicine and Medical Specialities, University of Alcala, 28801 Alcala de Henares, Spain; mademons@gmail.com; 4 Psikids, 28002 Madrid, Spain; isabel.morales@psikids.es

**Keywords:** disordered eating, eating disorders, eating behaviors, eating attitudes, anorexia nervosa, prevention

## Abstract

Background: current findings in the etiopathogenesis of eating disorders (ED) do not allow the formulation of a unique causal model. Currently, the main hypotheses about the etiopathogenesis are based on a multifactorial approach, considering both genetic and environmental factors. The aim of this study is to analyze the relationship between sociodemographic and behavioral factors, as well as self-esteem, in students of the first cycle of middle school and the probability of belonging to the risk group of eating disorders (ED) measured through the EAT-26 scale. Methods: The study target population consists of students of the first cycle of middle school. The instruments applied to the population consisted in: (1) a survey of sociodemographic data and behavioral variables; (2) Rosenberg’s self-esteem test; and (3) EAT Test (Eating Attitudes Test 26). Results: Of a total of 656 students belonging to eight educational centers in Madrid who were offered to participate in the study, 88.6% (*n* = 579) answered the whole questionnaire. The mean age of the participants was 13.7 years old. Of the participating adolescents, 57.3% were male and the remaining 42.7% (*n* = 260) were female. A significant relationship was observed between self-esteem and belonging to an ED risk group, with an OR = 0.910 (CI 95% 0.878–0.943). Hence, each one-point increase on the self-esteem dimension decreased the risk of belonging to an ED risk group by 9.5%. In the variables considered in the area of dysfunctional feeding patterns, the variables ‘number of meals’ (*p* < 0.01), ‘dieting’ (*p* < 0.01), and ‘drug consumption to lose weight’ (*p* < 0.01) were found to be related to the risk of belonging to the ED group. Conclusions: The results obtained in our research can help to establish explanatory models that include the understanding of the interaction of the different factors that influence the appearance and development of EDs. Therefore, these should be taken into consideration when developing ED preventive programs.

## 1. Introduction

Eating disorders are characterized by a disruption in normal eating habits leading to physical and psychological health deterioration [1]. Generally, eating disorders (EDs) are significantly related with the psychological, physical, and social aspects found in the daily life of the patients who develop this type of disorders [2,3]. A nationally representative survey of adolescents aged 13 to 18 years in the United States found that the lifetime prevalence of anorexia nervosa, bulimia nervosa, and binge eating was 0.3%, 0.9%, and 1.6% respectively [4]. Among them, anorexia and bulimia nervosa usually have a worse prognosis. They both share common clinical characteristics, such as excessive concerns about weight and body image, fear of gaining weight, and alterations in eating habits [5]. In addition, they have a distortion in their body shape with an extreme control of their diet, weight, and image [6]. Usually, individuals with EDs are not fully aware of their disease nor the risks associated with their behavior. Instead, they tend to focus all of their attention in losing weight, which causes nutritional deficiencies [6]. Diagnoses are based upon the American Psychiatric Association’s Diagnostic and Statistical Manual of Mental Disorders, Fifth Edition (DSM-5) [7].

The etiopathogenesis of EDs is multifactorial, considering both genetic and environmental factors [8,9]. It is one of the most common disorders during adolescence, which is also the period with higher vulnerability for personal and familial development [10,11]. Several environmental factors have been identified as leading to increased risk of developing EDs such as harmful childhood experiences (e.g., negligence, physical or sexual abuse, or parental dysfunction) or harmful experiences related with food and weight (e.g., family diet plan, overweight parents, negative comments about food, pressure to be slim from family, friends, etc.) [12,13,14]. Another important issue that has a strong relationship with ED pathological behaviors is the self-body image which acquires special importance during adolescence [10].

Several studies have suggested that both TV and new technologies use are implied in the appearance of the EDs [15,16]. In recent decades, the use of TV, video consoles, the internet, and social networks has multiplied, and curiously, the cases of anorexia eating disorders have also increased. Through the internet and social networks, young people expose themselves to an ideal of beauty that is difficult to achieve, which is sometimes a source of frustration [17]. In addition, this exposure of the body and the image that spreads through the internet and social networks invites people to makes comparisons, which is a known risk factor for developing ED, especially in adolescents with less self-esteem [18,19]. Eating patterns also play a relevant role in the etiopathogenesis of EDs. In this sense, previous studies have identified that dieting or skipping breakfast pose risk factors while eating with family or eating several meals a day and in a structured way protect against developing this type of disorder [20,21,22].

The aim of the present study is to analyze the relationship between self-esteem; eating habits, such as number of meals per day or the number of meals that the adolescents eat alone or with their families; weight loss behavioral patterns, such as starting a diet or taking drugs to lose weight; and the use of new technologies with the risk of developing eating disorders in an adolescent population. We consider it interesting and worthwhile to investigate the relationships of these factors among adolescents because they are the population most vulnerable to developing eating disorders [4]. Besides, there is evidence to suggest that attitudes, beliefs, and behaviors learned during these early years—for example, those relating to physical activity or food choices—show strong influence into adulthood [23]. In addition, mortality from eating disorders is high, around 5.1% in the case of anorexia nervosa and 1.7% in bulimia nervosa, mostly due to physical complications of the disease or increased risk of suicide [24]. Therefore, early detection is pivotal. Moreover, in people who already have symptoms of the disease, even if they do not meet the diagnostic criteria, cognitive behavioral therapy has shown the greatest effectiveness [25]. Finally, we hypothesize that those adolescents with lower self-esteem, greater tendency to skip meals, and increased use of new technologies are at greater risk of developing eating disorders.

## 2. Methods

### 2.1. Participants

The target population of this study consisted of middle school students (E.S.O.; in Spain includes adolescents from 12 to 16 years old) from eight different selected schools in Vallecas (Madrid, Spain). Within each selected school, sampling was conducted by clusters taking the classroom as a sample unit, until a representative sample of subjects was completed according to both their scholar courses, age, and sex. The participating classrooms of each school grade were randomly chosen. The evaluation was carried out in the schools. The application of the assessment instrument was always carried out collectively, only taking into account those students who were present in the classroom at that time, since the tutors were asked not to notify the group until the same day of application. Two of the trained evaluators were always present in each classroom in the absence of the teachers to prevent the students from feeling influenced when answering the questions. The duration of the test was approximately 45 min. 

### 2.2. Assessment Instruments and Variables Considered

We applied the following measurement instruments: (1) A questionnaire of sociodemographic data and behavioral variables of our own elaboration (Table 1); (2) Rosenberg’s self-esteem test; and (3) EAT-26 Test (Eating attitudes Test 26). The sociodemographic data questionnaire collected information regarding age, sex, school year (item 1), nationality (item 2), family life (item 3), academic performance (item 4), eating habits (items 5 to 11), physical exercise (items 12 and 13), and use of new technologies and media (items 14 to 23). Its purpose is to describe characteristics of the individuals studied. The Rosenberg’s self-esteem test is possibly the most widely used scale for measuring global self-esteem. It evaluates the perception that the subject has of him/herself. It consists of 10 items and a Likert-type response modality ranging from 1 (strongly disagree) to 4 (strongly agree). The classification of self-esteem is defined as high (>30 points), medium (20–30 points), and low (<20 points). In its original version, it has a reliability of 0.92 and an internal consistency of 0.72 [26]. The reliabilities for Spanish samples are above 0.70 [27]. On the other hand, the EAT-26 questionnaire is self-administered, and is considered one of the most universally accepted questionnaires for evaluating the risk of eating disorders with a reliability of 84% [28,29]. This test is used to determine the risk of an ED in educational institutions, universities, and other special risk groups [30,31]. The reliability of the EAT-26 questionnaire in Spanish samples is 92.1% in female and 0.89 in males [32,33]. Likewise, the Spanish version has shown a reliability of 0.80 in adolescent populations [34].

### 2.3. Ethical Considerations

The protocol was approved by the Clinical Research Ethics Committee of the Hospital General Universitario Gregorio Marañón in November 2009 (The ethical code number is 1775). The confidentiality of personal and medical data were maintained. All the students and their parents were informed of the study. Both the professor and a member of the research team provided written information regarding the study and its procedure and resolved any doubts that arose. After this, they gave their consent to participate. Students had the option of not participating or withdrawing their consent at any time. In case of feeling distressed by any question, the student could decide not to continue with the study.

### 2.4. Statistics

Descriptive and inferential statistical analyses were carried out. Continuous variables were described as means and standard deviations, and categorical variables as frequencies and percentages. Differences in sociodemographic variables, variables related to eating patterns, weight loss measures, variables related to new technologies and media use measures—according to eating disorder risk—were calculated using Student’s *t*-test (for continuous variables) or chi-square test (categorical variables) for statistical heterogeneity. We also used simple logistic regressions to calculate the probability of belonging to the risk group for ED per each increase of one point in the self-esteem dimension. These results were presented as odds ratio (OR) and their 95% confidence intervals (CI). Statistical significance was set at two-sided *p* of <0.05. These analyses were conducted with the software SPSS Statistics v23.0.0.0 (IBM Corp, Madrid, Spain).

## 3. Results

### 3.1. Descriptive Characteristics of the Participants

Of a total of 656 students belonging to eight educational centers in Madrid who were asked to participate in the study, 88.6% (*n* = 579) answered the whole questionnaire. The mean age of the participants was 13.7 years old. Of the participating adolescents, 57.3% (*n* = 319) were male and the remaining 42.7% (*n* = 260) were female; 69.3% (*n* = 379) of the subjects had Spanish nationality, while 30.7% (*n* = 168) had a foreign nationality. Among respondents, 79.9% (*n* = 445) of the subjects lived with both parents, 19.4% (*n* = 108) lived with only one of the parents, and 0.7% (*n* = 4) did not live with their parents. It was also found that 42.2% (*n* = 253) of the participants had repeated a school year, while 55.8% (*n* = 319) had never repeated a school year.

Concerning eating patterns, we found that 18.7% (*n* = 106) of the participants eat two or fewer meals, 32.6% (*n* = 186) eat three meals, 30.5% (*n* = 174) of the participants eat four meals, and 18.4% (*n* = 105) have five meals. Regarding the number of family meals, 4.7% (*n* = 26) do not have any family meals, whereas 40.3% had at least three meals with the family. Considering breakfast, 68.6% (*n* = 384) of the subjects have breakfast every day, whereas 8.2% (*n* = 46) reported never having breakfast.

Moreover, 22.9% (*n* = 130) of the participants had started a diet, while 76.9% (*n* = 436) had never started one. In fact, 3.4% (*n* = 19) had taken any kind of drug to lose weight. Regarding physical exercise, 39.8% (*n* = 158) exercised every day, 33% (*n* = 131) exercised one to two times a week, and 27.2% (*n* = 108) exercised more than three times per week.

In addition, regarding the use of new technologies and communication media, we found that 94.5% (*n* = 532) used the internet on a daily basis, 73.4% (*n* = 400) had a daily use of consoles, and 75.4% (*n* = 315) of the surveyed adolescents used a mobile phone every day. Regarding the use of TV, we found that 77.3% (*n* = 368) watched TV every day and 96% (*n* = 462) read fashion magazines every day.

Finally, the levels of self-esteem of the participants were high in 74.6% (*n* = 422), medium in 21.2% (*n* = 120), and low in 4.2% (*n* = 24), according to the Rosenberg self-esteem questionnaire.

### 3.2. Distribution of the Sample According to Eating Disorder Risk

The scores obtained in the EAT were transformed into a dichotomous qualitative variable defining the ‘risk of eating disorders’ as a score greater than or equal to 10, compared to ‘no risk’, with scores lower than 10. Table 2 and Table 3 show the distribution of the dichotomotic variable ‘risk/no risk of ED’.

In the age variable, no statistically significant differences were observed between those subjects who belong to the ED risk group and those who do not belong to the ED risk group (*p* = 0.356) (Table 2). In the sex variable, no statistically significant differences were found (*p* = 0.254) based on belonging to the ED risk group/non-ED risk group (Table 2). Nationality also did not show a statistically significant relationship with the variable risk of eating disorders/no risk of eating disorders (*p* = 0.256) (Table 2). The last sociodemographic variable is family coexistence, and we also found no relationship between this variable and belonging to the risk/non-risk group of eating disorders (*p* = 0.483) (Table 2).

Regarding educational variables, the relationship between repeating an academic year and the risk of developing eating disorders was assessed, finding no statistically significant relationship between these variables (*p* = 0.148) (Table 3). In the variables considered within the area of dysfunctional eating patterns, ‘number of meals’ (*p* < 0.01), ‘dieting’ (*p* < 0.01), and ‘using drugs to lose weight’ (*p* < 0.01) are related to the variable risk of eating disorders. While the variables ‘at least 3 meals a day’ (*p* = 0.072), ‘having breakfast’ (*p* = 0.363), ‘exercise’ (*p* = 0.307), and ‘exercise frequency’ (*p* = 0.225) did not show statistically significant relationships with the risk variable for eating disorders (Table 3).

Regarding the use of new technologies and the media, ‘Internet frequency’ (*p* < 0.01), ‘mobile frequency’ (*p* < 0.01), ‘fashion magazines’ (*p* < 0.01), ‘fashion magazine frequency’ (*p* < 0.01), and ‘use of game consoles’ (*p* < 0.05), a statistically significant relationship was found with the risk variable for eating disorders. The variables ‘Internet use’ (*p* = 0.484), ‘mobile phone use’ (*p* = 0.497), ‘frequency of use of game consoles’ (*p* = 0.102), ‘use of TV’ (*p* = 0.626), and ‘frequency of TV use’ (*p* = 0.509) did not show statistically significant relationships with the risk variable for eating disorders (Table 4).

### 3.3. Self-Esteem as a Predictor of Eating Disorder Risk

Subjects with higher self-esteem (measured through the Rosenberg Scale) have a lower risk of developing ED (measured through the EAT-26 test). Per each increase of one point in the self-esteem dimension, the risk of belonging to the risk group for eating disorders was reduced by 9.0 % (OR = 0.91; 95% CI 0.88–0.94: *p*-value < 0.001).

## 4. Discussion

The present work focuses on describing the role that self-esteem, eating, and health habits, as well as the patterns of use of new technologies and fashion magazines play in the development of eating disorders in adolescents. Sociodemographic and academic variables (age, sex, nationality, or academic failure) have not shown a statistically significant relationship with being in the group of subjects at risk for developing eating disorders. Regarding the variables related with eating habits and weight loss behaviors, the number of meals, dieting, and the use of weight loss drugs were statistically significantly related with belonging to the risk group. On the other hand, the variables related to the use of new technologies and social influence that have shown a statistically significant relationship with belonging to the risk group have been the frequency of internet use, the frequency of mobile use, the use of fashion magazines, the frequency of use of fashion magazines, and the use of game consoles. Finally, the subjects with higher self-esteem had a lower risk of having an ED.

Early adolescence has been considered a particularly relevant period for the formation and development of self-esteem, in which individuals are more vulnerable to experiencing a decrease in their own self-esteem. This stage is characterized by the experience of novel and sometimes stressful events that challenge adolescents’ vision of themselves and their emotional stability [35,36]. Thus, young people at these ages frequently show, not only a decrease, but also strong fluctuations in their levels of self-esteem, which tend to decrease as adolescence progresses and adulthood is reached [37,38]. In previous studies, it has been observed that both the decline and fluctuations in self-esteem that occur in early adolescence are connected with significant negative experiences such as academic difficulties or loss of support from peers [39]. Adolescents with lower self-esteem are in turn more vulnerable to the impact of everyday events than those with higher self-esteem [40]. In addition, low self-esteem has been considered a relevant factor of vulnerability for the development and maintenance of eating disorders [41,42]. Self-esteem is considered as a prior, predisposing factor, and as a later symptom of eating disorders [43,44]. This means that self-esteem not only acts as a factor closely related to the disorder emergence but can also be considered as an influencing variable in the course of the disease. 

In our sample, both age and sex did not show a statistically significant relationship with the variable belonging to the risk group for developing eating disorders. Despite the fact that most of the studies published so far regarding sex indicate that women are more vulnerable to this disorder, men are not exempt from presenting them, although in a lower percentage [45]. However, a nationally representative survey of adolescents (aged 13 to 18 years) in the United States found that the lifetime prevalence of anorexia nervosa was similar for females and males [4]. Interestingly, in our research, the sex variable did not show a statistically significant relationship with the risk variable for developing ED, with a similar distribution between men and women. Until now, the main explanation as to why women are more prone to develop an eating disorder is the result of social pressure to fulfil an ideal of thinness, especially in the age of social media [46,47]. However, the evolution in recent years of the sociocultural context has made men a target of eating disorders as well [48]. Thinness and beauty standards are becoming more and more demanding in men, approaching the extremes of women. Men increasingly tend to take care of their body image and have also become victims of advertising [49]. In the specific case of current adolescents, the internalization of beauty canons could be carried out from this perspective, in which social pressure regarding thinness is equalized between sexes.

Numerous studies have already pointed out the importance of the use of new technologies in the appearance and maintenance of EDs [50,51]. On the other hand, exposure to the media seems to precede the emergence of eating disorders, since ideas, models, and innumerable products and services that do not reproduce common women or men, nor represent a healthy body weight are disseminated on TV [52]. In a longitudinal study carried out a few years ago, it was found that a longer time of exposure to television programs predicted the internalization of the ideals of appearance and thinness one year later [53].

Different studies have pointed out dieting in adolescents is a risk factor for developing eating disorders [54,55]. Inappropriate behaviors for weight control have also been associated with negative outcomes related to eating disorders in the subsequent 5 years [56]. Finally, it is worth noting that in our sample only 30.5% of the subjects had four meals a day. These values obtained in our study are concerning, since the voluntary omission of daily intakes could be a triggering factor of an ED [57].

However, our work has limitations as it was designed as a cross-sectional study, therefore causality cannot be inferred. In addition, the questionnaires we have used have limitations. For example, repeating a school year is not a comprehensive indicator of school performance. With regard to the use of new technologies, we did not explore what use the adolescents made of new technologies, we only made a quantitative record. Finally, some words such as ‘diet’ could be interpreted differently by different people. Nevertheless, we have included a high number of participants that were homogenous in nature. Future studies should include follow-up over time in order to determine not only the role that these sociodemographic, familiar, eating pattern, and use of new technologies variables have on the risk of developing ED, but they should also investigate what role they play in the symptomatology and evolution of the disease.

## 5. Conclusions

Self-esteem acts as a key factor in increasing the likelihood of developing an eating disorder. Furthermore, we have found an inverse correlation between self-esteem and the risk of developing an ED, as an increase of one point in the self-esteem dimension, decreased the probability of belonging to the group at risk of developing an eating disorder by 9.5%. The use and frequency in which the adolescents in our sample used the different media (TV, fashion magazines, and the Internet) increased the probability of belonging to the group at risk of developing an eating disorder. In relation to the variables related to eating patterns, dieting during adolescence significantly increased the probability of belonging to the risk group for developing an eating disorder. The results obtained in our research can help to establish preventive and or early intervention programs in eating disorders. Promoting health has long been an important role of schools, but traditionally activities have focused on health education. However, according to our results it would be beneficial to include activities and tools aimed at improving the self-esteem of adolescents.

## Figures and Tables

**Table 1:** Sociodemographic data and behavior variables questionnaire.

(1) Age: - Sex: Female/Male - School year:
(2) Spanish nationality: Yes/No Other (Which?):__________ (3) Living with: (Circle what applies): Father, mother, Father’s couple, Mother’s couple, siblings, others. (4) Schooling data: Have you ever repeated a year? YES NO–Which one(s)?____ **Eating habits:** Circle what applies: Do you eat? In family? (5) Breakfast: YES/NO/ Sometimes; YES/NO (6) Midmorning: YES/NO/Sometimes; YES/NO (7) Lunch: YES/NO/Sometimes; YES/NO (8) Afternoon snack: YES/NO/Sometimes; YES/NO (9) Dinner: YES/NO/Sometimes; YES/NO (10) Have you ever been on a diet? YES/NO (11) Have you ever taken drugs to lose weight? YES/NO **Physical exercise:** (12) Do you do any kind of physical exercise outside of school hours? YES/NO (13) How often? Every day/1 to 2 times per week/≥3 times per week **Use of new technologies and communication media:** Do you use the following?: YES/NO How often?: 0–2 hours/day, 2–4 hours/day, >4 hours/day? (14)(15) Internet (16)(17) Cell phone (18)(19) TV (20)(21) Consoles (22)(23) Indicate if you read fashion magazines (Yes/No) and how often you do it (Circle what applies) c: 0–2 h/day, 2–4 hours/day or >4 h/day

**Table 2:** Distribution of sociodemographic variables according to eating disorder risk.

	At Risk of ED	No Risk of ED	Test Statistics	*p*-Value
**Sociodemographic variables**				
**Age**, mean (SD) **Sex**, *n* (%) Women Men	13.77 (0.90) 146 (65.8%) 215 (70.7%)	13.67 (0.83) 76 (34.2%) 89 (29.3%)	t = 0.85 χ^2^ = 1.465	0.356 0.254
**Nationality**, *n* (%)				
Spanish	103 (28.9%)	253 (71.1%)	χ^2^ = 1.312	0.256
Foreign	54 (34.0%)	105 (66.0%)		
**Family coexistence**, *n* (%)				
With parents	295 (81.0%)	126 (76.8%)	χ^2^ = 1.457	0.483
With one parent	66 (18.1%)	37 (22.6%)		
Without parents	3 (0.8%)	1 (0.6%)		

**Table 3:** Distribution of variables related to eating patterns and weight loss measures according to eating disorder risk.

	At Risk of ED	No Risk of ED	Test Statistics	*p*-Value
**Academic**				
**Academic performance**, *n* (%)				
Yes	79 (47.6%)	153 (40.9%)	χ^2^ = 2.094	0.148
No	87 (52.4%)	221 (59.1%)		
**Eating Pattern**				
**Number of meals***n* (%)				
2	39 (33.5%)	62 (16.6%)	χ^2^ = 22.650	0.001
3	69(41.6%)	112 (29.9%)		
4	33 (19.9%)	127 (34.0%)		
5	25 (15.1%)	73 (19.5%)		
**At least 3 meals per day**				
Yes	39 (46.5%)	62 (16.6%)	χ^2^ = 3.617	0.072
No	127 (76.5%)	312 (83.4%)		
**Breakfast**				
Yes	105 (65.2%)	256 (69.6%)	χ^2^ = 2.027	0.363
No	17 (10.6%)	26 (7.1%)		
Sometimes	86 (23.4%)	86 (23.4%		
**Diet**				
Yes	66 (53.2%)	58 (46.8%)	χ^2^ = 39.382	0.001
No	98 (23.8%)	314 (76.2%)		
**Weight-loss drugs**				
Yes	4 (1.1%)	12 (7.3%)	χ^2^ = 14.862	0.001
No	362 (98.9%)	153 (92.7%)		
**Exercise**				
Yes	49 (29.7%)	104 (28.3%)	χ^2^ = 2.359	0.307
No	115 (69.7%)	263 (71.7%)		
**Exercise frequency**				
Everyday	52 (44.4%)	94 (35.9%)	χ^2^ = 2.985	0.225
1–2 times/week	33 (28.2%)	94 (35.9%)		
3 or more times/week	32 (27.4%)	32 (27.4%)		

**Table 4:** Distribution of variables related to new technologies and media use measures according to eating disorder risk.

	At Risk of ED	No Risk of ED	Test Statistics	*p*-Value
**Internet**				
Yes	157 (95.2%)	349 (94.6%)	χ^2^ = 0.075	0.484
No	8 (4.8%)	20 (5.4%)		
**Internet frequency**				
0–2 h/day	59 (42.4%)	199 (62.6%)	χ^2^ = 20.170	0.001
2–4 h/day	38 (27.3%)	73 (23.0%)		
4 or more hours/day	42 (30.2%)	46 (14.5%)		
**Mobile phone**				
Yes	148 (89.2%)	316 (85.9%)	χ^2^ = 1.398	0.497
No	18 (10.8%)	51 (13.9%)		
**Mobile phone frequency**				
0–2 h/day	62 (48.1%)	176 (64.7%)	χ^2^ = 10.913	0.004
2–4 h/day	24 (18.6%)	41 (15.1%)		
4 or more hours/day	43 (33.3%)	55 (20.2%)		
**Video game console**				
Yes	110 (28.9%)	271 (71.1%)	χ^2^ = 6.473	0.039
No	52 (38.5%)	83 (61.5%)		
**Video game console use**				
0–2 h/day	60 (26%)	171 (74%)	χ^2^ = 4.563	0.102
2–4 h/day	26 (39.4%)	40 (60.6%)		
4 or more hours/day	11 (31.4%)	24 (68.6%)		
**Fashion magazines**				
Sí	69 (45.4%)	87 (28.0%)	χ^2^ = 16.291	0.001
No	82 (53.9%)	224 (72.0%)		
**Fashion magazines use**				
0–2 h/day	80 (52.6%)	224 (72.3%)	χ^2^ = 22.165	0.001
2–4 h/day	57 (37.5%)	78 (25.2%)		
4 or more hours/day	15 (9.9%)	8 (2.6%)		

## Data Availability

The data that support the findings of this study are available from the corresponding author upon reasonable request.
